# FSH and TSH in the Regulation of Bone Mass: The Pituitary/Immune/Bone Axis

**DOI:** 10.1155/2013/382698

**Published:** 2013-05-30

**Authors:** Graziana Colaianni, Concetta Cuscito, Silvia Colucci

**Affiliations:** Department of Basic Medical Sciences, Neuroscience and Sense Organs, Section of Human Anatomy and Histology, University of Bari, Piazza Giulio Cesare 11, 70124 Bari, Italy

## Abstract

Recent evidences have highlighted that the pituitary hormones have profound effects on bone, so that the pituitary-bone axis is now becoming an important issue in the skeletal biology. Here, we discuss the topical evidence about the dysfunction of the pituitary-bone axis that leads to osteoporotic bone loss. We will explore the context of FSH and TSH hormones arguing their direct or indirect role in bone loss. In addition, we will focus on the knowledge that both FSH and TSH have influence on proinflammatory and proosteoclastogenic cytokine expression, such as TNF**α** and IL-1, underlining the correlation of pituitary-bone axis to the immune system.

## 1. Introduction 

Bone undergoes remodeling throughout life, and this process is orchestrated by a variety of cytokines and hormones which play a critical role in skeletal homeostasis in health and disease. 

The major evidence showing the link between hormone levels and altered bone remodelling came from several studies demonstrating the key role of estrogens in bone turnover, in both women and men [1]. However, over the past ten years, longitudinal studies have related the pituitary hormones levels to measurements of bone microstructure and bone turnover markers, across the menopause transition [2]. In fact, the pituitary-bone axis is being widely recognized, in view of its function as endocrine skeletal regulation, particularly in the context of osteoporotic bone loss. Accordingly, ongoing evidences have proved that some pituitary hormones play an important role in bone regulation, such as growth hormone (GH) [3], follicle stimulating hormone (FSH) [4], thyroid stimulating hormone (TSH) [5], prolactin (PRL) [6], and oxytocin [7]. Furthermore, experiments performed in haploinsufficient mice for pituitary hormones or their receptors showed that the decline of hormone levels leads to altered bone structure and dynamics while the primary target organ remains unaffected, highlighting that bone could be even the more sensitive organ to the pituitary hormone effects [7]. 

This finding resulted in a reassessment of the paradigm according to which the decline of estrogen levels is responsible for bone loss during menopause and aging. This assumption came from the evidence that increased bone loss could be also attributed to the high FSH serum levels during the menopause transition [2], a condition in which estrogen serum levels are still normal.

Notably, osteoporosis has also been associated to thyroid dysfunction in older women, as the risk for fracture was associated with low serum levels of TSH. These results addressed the proposal that TSH was a key negative regulator of bone turnover and that bone loss was a consequence of TSH deficiency rather than thyroid hormone excess [5].

Based on these and much other knowledge, in the following paragraphs we discuss the role of FSH and TSH and their relevance in the alterations of the pituitary-bone axis leading osteoporosis and imbalance in bone homeostasis.

## 2. FSH

It has long been considered that the primary specific action of follicle-stimulating hormone (FSH) is to stimulate ovarian folliculogenesis and estrogen synthesis. Despite that FSH has a well-established role in reproduction, a controversial issue regarding the association of its high circulating levels and bone loss has emerged [8]. The issue came from studies involving pre- and perimenopausal women showing that elevated serum concentrations of FSH correlate or do not with bone mineral density (BMD) or bone resorption markers even before menopause and decline in estradiol [8–10]. Moreover, a meta-analysis of ten prospective studies revealed that the rate of spinal BMD loss during perimenopause, when estrogen levels were still high, was greater than the rate of loss in the years following menopause, when estrogen levels were much lower [11].

Direct evidence for FSH modulation of osteoclast differentiation has been provided in mouse and human cells [12], which have pointed out a role of FSH in the menopausal bone loss. The authors showed that osteoclastogenic and proresorptive actions of FSH are exerted through a G_*i*2*α*_-coupled FSH receptor (FSHR) that has been identified on both human and mouse osteoclast and their precursors [12]. In osteoclast, FSHR activation enhances the phosphorylation of downstream RANKL sensitive kinases, Erk (extracellular signal-regulated kinases), Akt and I*κ*-B*α*, an inhibitor of NF-*κ*B (nuclear factor kappa-light-chain-enhancer of activated B cells) to transduce the proresorptive actions of RANK-L [12]. To provide genetic evidence about the effect of FSH on the skeleton, that was exerted independently of estrogen, the authors used mice lacking the β-subunit of FSH or the FSH-Receptor and showed that these mice resulted in having high bone mass although they had normal levels of estrogen [12]. Notably, the authors found that this high bone mass was related to reduced bone resorption, as revealed by histomorphometry and ex vivo cultures of bone marrow cells [12]. The enhanced bone mass and osteoclast defect in eugonadal, but FSH haploinsufficient mice, separated the skeletal actions of FSH from those of estrogen. Moreover, hypogonadal FSHβ−/− and FSHR−/− mice also failed to lose bone despite their severe deficiency of estrogen [12]. In contrast to this, another work has shown that FSHR−*/*− mice have, however, an age-dependent loss of bone mass, which is further reduced upon androgenic decline, demonstrating that the androgen withdrawal, as well as the estrogen (androgen-derived via aromatase action) withdrawal, has an inhibitory effects on bone formation and an even more evident positive effect on bone resorption [13]. Moreover, loss of FSH signaling in FSHβ−*/*− and FSHR−*/*− mice causes the expected loss of ovarian aromatase production, by reducing ovarian estrogen production [14]. The authors underlined the observation that the ovaries were not completely hormone deficient since, in these null mice, the levels of testosterone, known to be anabolic for bone, were 10-fold higher than wild-type mice due to increased LH [14]. These data suggest that the elevated testosterone contributed to skeletal maintenance of bone mass in the FSHR null mice [15–18]. 

A lack of a direct effect of FSH on bone in mice has recently been supported by a study that showed neither daily injections of FSH nor continuous infusion of FSH for 1 month on male mice had an effect on femoral bone mineral density [19]. The same authors, in contrast to data from Sun et al. [12], demonstrated that osteoclastogenesis from both human mononuclear cell precursors and RAW 264.7 cell line was not affected by FSH [19]. Although these opposite in vitro findings remain to be better elucidated, a recent explanation of contrasts for the in vivo results emerged in the study of Gourlay et al. [20]. In particular a cross-sectional study of postmenopausal women aged 50 to 64 showed that FSH was independently associated with lean mass but not BMD, suggesting that the correlations between FSH and BMD might reflect weight-bearing and nonweight-bearing effects of greater lean mass and weight on BMD [20].

Consistent with the panel of studies showing the FSH-independent role in menopausal bone loss, another human clinical analysis of Drake et al. [21] has demonstrated that suppression of FSH secretion in postmenopausal women, using a GnRH agonist, did not reduce levels of bone resorption markers [21]. 

In this complex scenario, the work of Allan et al. should be considered [22], who created a pituitary-independent mouse model using a transgenic expression of human FSH (TgFSH) in female mice, to better investigate the role of FSH in regulating bone loss. This study reveals that elevated FSH activity in vivo markedly stimulates bone mass via an ovary-dependent pathway. Despite the questionable use of this chimera mouse, this study highlights the positive association of FSH-induced ovarian secretion of testosterone and inhibins, which in turn suppress pituitary FSH secretion, with elevated bone mass and the absence of direct FSH stimulatory actions on bone [22].

The FSH effects on bone also emerged in some genetic studies, in which the severity of menopausal bone loss has also been linked to polymorphisms in the FSHR gene. Women with the genotype AA rs6166 may undergo osteoporosis with a significantly higher incidence of those with the GG rs6166 allele, independently of serum FSH or estrogen levels [23]. This result clarifies the genetic trend to develop osteoporosis and might explain the reason why although estrogens has anabolic [24, 25] and antiresorptive actions [26, 27], the bone resorption that occurs during late perimenopause, when estrogen levels are normal, could be correlated to the changes in FSH levels.

In addition to all these data, it is important to underline that the investigation regarding the FSH effects on bone has also been correlated to the immune cell alterations occurring during perimenopause. In fact, T lymphocytes and inflammatory cytokines, such as TNF-*α* and IL-7, are strongly involved in hypogonadal bone loss [28]. The relevance of TNF-*α* in the increased osteoclast formation during menopause has been demonstrated by several animal models. TNF-*α* null mice or p55-TNF-Receptor null mice were unable to induce bone loss after ovary surgically ablation [28]. Furthermore, the treatment with TNF-*α* inhibitor protects from ovariectomy-induced bone loss [29]. Elevated levels of TNF were found in bone marrow of ovariectomy mice [30] and in the conditioned media of peripheral blood mononuclear cells from postmenopausal women [31].

In this respect Iqbal et al. have proposed that the effects of FSH on bone mass are, at least in part, exerted via the modulation of TNF*α* production by bone marrow macrophages and granulocytes demonstrating the inhibition of bone loss in FSHβ-deficient mice derived from decreased TNF*α* production [32]. The study draws on the observation that FSHβ-deficient mice have lower serum levels of TNF*α* compared with littermate controls suggesting that the lack of FSH signaling could counteract the opposite stimulatory effect on TNF*α* production after estrogen decline. Based on their in vitro studies, the authors have also reported an increase in supernatant TNF-*α* levels upon the exposure of bone marrow cultures to recombinant FSH. This TNF*α* synthesis resulted from macrophages-granulocytes (CD11b), but not from B lymphocytes (B220) and T lymphocytes (CD3) [32]. 

The involvement of immune cytokines regulating the bone-resorbing activity of osteoclasts has extended the research of FSH-mediated effects on interleukins secretion. Based on these observations, Cannon et al. hypothesized that FSH influences BMD, in part, by affecting the activity of bone-resorbing cytokines, either by inducing their secretion or by altering their receptor expression [33]. Thirty-six women between the ages of 20 and 50 were enrolled for bone mineral density analysis, cytokine ligand and soluble receptor concentrations, and surface expression of cytokine receptors on monocytes. Isolated peripheral blood mononuclear cells from these subjects, when incubated with exogenous FSH, increased the secretion of IL-1β, TNF-*α*, and IL-6 in proportion to the surface expression of FSH receptors on the monocytes. Endogenous FSH concentrations, measured in serum from these women, were found to be proportional with the circulating concentrations of these cytokines. None of these individual cytokines was related to BMD, unless for the IL-1β-to-IL-1 receptor antagonist (IL-1Ra) ratio that was found to be inversely related to BMD [33]. 

Furthermore in a more recent study performed on human subjects, Cannon et al. have also proposed that FSH promotes the receptor activator for NF-kB (RANK) expression on human monocytes (CD14+) [34]. In particular the authors found no significant influence on RANK expression when peripheral blood mononuclear cells were treated with FSH at a concentration of 10 mIU/mL, which is in the range of FSH in the follicular phase of women during the majority of their reproductive years. However, when FSH was used at 50 mIU/mL, they found a significant increase in RANK expression. This concentrations reflect the common FSH levels during perimenopause associated with increased bone loss. At higher concentrations reached after menopause (100 mIU/mL), FSH had less influence on RANK expression, according to clinical observations showing that the loss of spinal BMD is greater during perimenopause than in the years following menopause [34].

Having identified several evidences showing that bone loss occurring during perimenopause is in part FSH related, the prevention of menopausal osteoporosis could be done in advance: women, thus, can be screened earlier for bone loss, basing the first diagnosis on their FSH levels. The diagnosis of osteoporosis is performed through the measurement of T-scores, which compare the patient BMD against a database of young, 30-year-old Caucasian women [35]. The microarchitectural deterioration caused in part by FSH during late perimenopause, in the form of trabecular perforations [36] does not decrease the T-score as in the osteoporotic range; even it can decrease the bone strength [37]. Thus, even if the BMD may be normal, the increased bone fragility during late perimenopause would not be diagnosed. This means the need to develop and utilize other methods of screening, over BMD, which have a greater precision also for the detection of fragility. This could be translated into earlier diagnosis and, in particular, in new therapies for menopausal bone loss. A proposed treatment has been suggested by Zhu et al., who generated and characterized a polyclonal antibody to a 13-amino-acid-long peptide sequence within the receptor-binding domain of the FSH β-subunit [38]. The authors showed that the FSH antibody binds FSH specifically and blocks osteoclast formation in vitro. Experiments on ovariectomized mice confirmed the attenuation of bone loss after FSH antibody injection not only by inhibiting bone resorption but also by stimulating bone formation [38]. Thus, mesenchymal cells isolated from mice treated with the FSH antibody had greater osteoblast precursor, in a similar extent of mesenchymal cells isolated from FSHR−/− mice [38]. Consistent with this new finding, FSH negatively regulates osteoblast number and this suggests that the FSH-blocking agent could be a new therapy to restore the uncoupling between bone formation and bone resorption which occurs in several bone loss associated diseases.

## 3. TSH

Thyroid stimulating hormone (TSH) has been claimed to regulate solely the thyroid follicular growth and the thyroid hormone secretion by binding to a seven transmembrane, glycosylated G protein-coupled receptor, and the TSH receptor (TSHR) on the thyroid gland. Afterwards new studies had identified TSHRs in other tissues and cells, including the pituitary, thymus, testes, kidney, brain, lymphocytes, adipocytes, and fibroblasts [39, 40], but their functional significance has remained unclear. Regarding the bone tissue, the high-turnover osteoporosis in hyperthyroidism has been attributed solely to elevated thyroid hormones. Interestingly, however, the therapeutic suppression of TSH or subclinical pathology of hyperthyroidism, in which TSH is low and thyroid hormones are normal, are both associated with profound osteoporosis suggesting a direct antiresorptive role of TSH [41, 42]. This indicates that TSH acts on bone independently of thyroid hormones and that the osteoporosis of hyperthyroidism is, at least in part, due to low TSH levels.

Over recent years, new evidences have emerged showing that TSH exerts direct effects on skeletal remodelling by interacting with specific receptors expressed on bone cells [43]. In experimental animals, reduced expression of TSH receptor led to the osteoporosis development, inhibiting bone turnover [44]. Moreover, administration of low doses of TSH in ovariectomized rats improved bone microstructure and prevented osteoporosis [45].

Studies on TSHR−/− mice show evidence of increased osteoclastic activity, as well as hyt/hyt mice, which have defective TSHR signaling [46]. This mouse model has definitively clarified the direct antiosteoclastogenic action of TSH which acts on its receptor inducing a reduction in NF-*κ*B and JNK signaling and in TNF-*α* production [45, 47]. It has recently been demonstrated that the effect of TSH on TNF-*α* synthesis is mediated transcriptionally by binding of two high mobility group box proteins, HMGB1 and HMGB2, to the promoter of the TNF-*α* gene [48].

As expected, TNF-*α* production is upregulated in TSHR−/− mice, which increases osteoclastic activity and contributes to the osteopenic phenotype [44]. To further corroborate these findings, the genetic deletion of TNF-*α* in TSHR−/− mice was studied and bone resorption was found to be reversed [46]. These authors found that osteoporosis in TSHR knockout mice was the result of an enhancement in osteoclast differentiation. Consistent with the low bone mass, ex vivo cultures of bone marrow cell precursors from both heterozygote and homozygote mice showed increased osteoclast formation and the enhanced expression of an osteoclast marker tartrate-resistant acid phosphatase (TRAP). This enhanced osteoclast formation was dependent on a several-fold increase in the synthesis and release of TNF*α*. A blocking antibody to TNF*α* abrogated this increased osteoclastogenesis, suggesting that osteoporosis in the TSHR−/− mice was TNF*α* mediated [46]. Furthermore, although all three cytokines, TNF*α*, IL-1, and IL-6, were elevated in TSHR−/− cultures, only TNF*α* was elevated in the cultures from heterozygote mice, indicating a dominant effect of TSH on TNF*α* secretion. The authors also examined whether, in the TSHR genotypes, there were significant differences in the populations of immunosystem cells, such as macrophages (CD11b^+^), leukocytes (CD45^+^), T lymphocytes (CD8^+^, CD90^+^, CD4^+^, CD3^+^), and B cells (B220^+^). The investigators showed that CD11b^+^ and CD45^+^ precursor populations were significantly increased in both TSHR−/− and TSHR−/+ mice, whereas the B220^+^ cell population was reduced. The hypothesis that TSH acts solely through CD11b^+^ osteoclast progenitors was attested demonstrating that TSH attenuated cytokine-induced TNF*α* mRNA and protein expression only in CD11b^+^ cells through AP-1 and NFkB activation via transcriptional effect [46]. This finding increased the hypothesis that osteoporosis in human hyperthyroidism may also be TNF driven.

In postmenopausal women, a single subcutaneous injection of recombinant human TSH drastically lowers serum C-telopeptide, as marker of bone resorption, to premenopausal levels within two days, with recovery at day 7 [49]. The effects of recombinant TSH in clinical studies showed a reduction in serum C-telopeptide levels [49] although the effect on serum RANKL and OPG is not clear yet. In a study by Giusti et al. [50], the authors found no alteration in these cytokines expression in response to recombinant TSH in patients receiving L-thyroxine for the treatment of thyroid carcinoma. Martini et al. [51], however, have reported an increase in type-1 procollagen N-terminal propeptide (PINP), a marker of bone formation. This validates the conclusion drawn from previous studies revealing that TSH could also have anabolic action [52].

The sensitivity of the adult skeleton to altered thyroid status is illustrated by the reduction in BMD and the increase in fracture risk in postmenopausal women and men with subclinical hyperthyroidism. Despite this evidence, although a number of studies have suggested that TSH may directly inhibit bone turnover, other studies are still conflicting and the anabolic role of TSH was not clarified yet. This important questions will be resolved using conditional mouse targeting specifically TSH in osteoblast and osteoclast to identify which bone cells are directly responsive in vivo.

## 4. Conclusions

**Figure 1:**
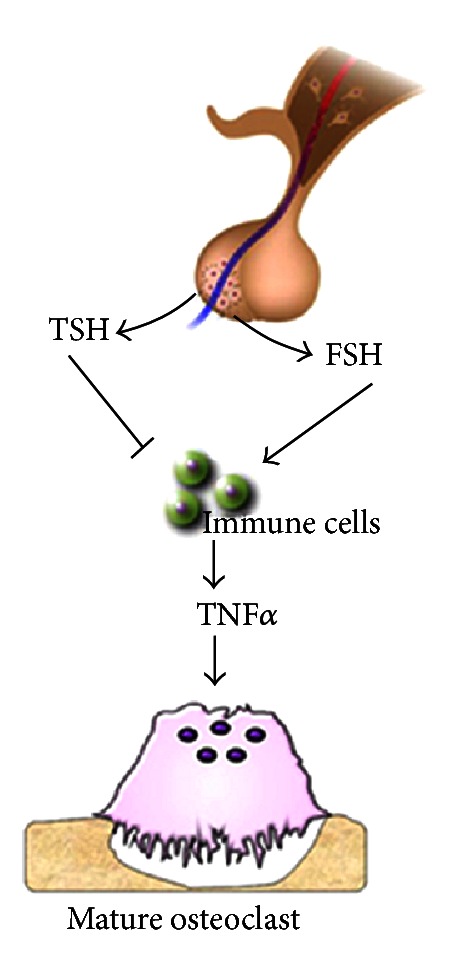
Scheme of pituitary-bone axis. FSH and TSH effects on bone turnover via TNF*α* secretion by cells of immune system.

In the overview of the pituitary-bone axis actions on bone metabolism, although several elegant in vivo and in vitro studies have been performed in human and murine animal models to investigate FSH effects on bone, this issue still remains controversial. Some authors showed a positive direct or indirect FSH effect on osteoclast formation and function, while other investigators do not find any role of the hormone on the skeleton. Thus, the topic of FSH actions on bone needs to be better resolved. Conversely, no conflicting data have emerged in the investigation of TSH effect on bone due to the existence of consistent results demonstrating its role in inhibiting bone turnover. Moreover, in the last decade many investigators have emphasized the importance of the immune cytokines as key regulators of bone metabolism. With particular regard to the role of FSH and TSH on bone remodelling, it has been reported that TNF*α* could be the mediator of hormone effects (Figure 1). The understanding of this and/or other intermediating molecule/s in the FSH and TSH signalling could have a great importance in early diagnosis and better management of pituitary hormone-dependent bone loss.
